# The complete mitochondrial genome of *Lyssa zampa* (Lepidoptera: Uraniidae)

**DOI:** 10.1080/23802359.2021.1915715

**Published:** 2021-05-31

**Authors:** Ge-Ge Yuan, Yuan-Wen Du, Lu Chen, Lang Ming, Gong Chen, Xing Wang

**Affiliations:** a College of Plant Protection, Hunan Agricultural University, Changsha, China; b Hunan Provincial Key Laboratory for Biology and Control of Plant Diseases and Insect Pests, Hunan Agricultural University, Changsha, China

**Keywords:** *Lyssa zampa*, mitochondrial genome, phylogenetic position

## Abstract

The complete mitochondrial genome (mitogenome) of *Lyssa zampa* was first reported. It is 15,314 bp in length (GenBank accession number: MW435592) and consists of 13 protein-coding genes (PCGs), 22 transfer RNA (tRNA) genes, two ribosomal RNA (rRNA) genes. The nucleotide composition is A (41.5%), C (11.1%), G (7.4%), and T (40.0%). Based on the sequences of complete mitogenome from 12 geometroid species and three drepanoid species as ingroups, and two noctuoid species as outgroups, the phylogenetic tree was constructed. The results showed that the closest relationship between Uraniidae and Epicopeiidae was strongly supported by Bayesian posterior probabilities values of 0.99.

*Lyssa zampa*, which is broadly distributed from the Himalayas to Borneo and the Malay Peninsula, belongs to the family Uraniidae (Lepidoptera: Geometridae) (Nazari et al. 2016). Its larvae feed on *Endospermum* and other members of the rubber tree in the family Euphorbiaceae (Tokeshi and Yokoo 2007), and its genetic information is rarely known. Here, the complete mitochondrial genome (mitogenome) of *L*. *zampa* was first obtained and reported in the family Uraniidae, and the phylogenetic tree for understanding its phylogenetic position and relationships within the superfamily Geometridae was constructed. All the specimens and the genomic DNA in this study were deposited at the Insect Museum of Hunan Agricultural University, Changsha City, Hunan Province, China.

The specimens of *L*. *zampa* were collected on 21 July 2019 from Xiajinchang Town (23°N, 105°E, elevation of 1395 m) in Malipo County, Yunan Province, China. The complete genomic DNA was extracted following the methods in Wang et al. (2019) from the legs of the adult (collection number: HAUHL033878) by using TaKaRa MiniBEST Universal Genomic DNA Extraction Kit Ver.5.0 (Shiga Prefecture, Kusatsu City, Japan), and was sequenced on the Illumina Hiseq platform with 150 bp paired-end reads at Novogene (Beijing, China). For Illumina TruSeq library, 6 Gb of clean data were obtained. The contigs and scaffolds of highly qualified sequences were determined using IDBA-1.1.3 (Peng et al. 2012; Chen et al. 2020). The mitogenome was annotated on the ORF finder (https://www.ncbi.nlm.nih.gov/orffinder/) and MITOS web server (http://mitos.bioinf.uni-leipzig.de/index.py) (Bernt et al. 2013).

The complete mitogenome of *L*. *zampa* is a circular DNA molecule of 15,314 bp in length (GenBank accession number: MW435592) and consists of 13 protein-coding genes (PCGs), 22 transfer RNA (tRNA) genes, two ribosomal RNA (rRNA) genes, and an AT-rich region, in which 23 genes are transcribed on the J strand and the remaining 14 are transcribed on the N strand. The nucleotide composition is A (41.5%), C (11.1%), G (7.4%), T (40.0%), and the AT nucleotide content is 81.5%. There are 76 bp intergenic nucleotides that were dispersed in between 14 pairs of neighboring genes with their length varying from 1 to 24 bp. The length of the AT-rich region that located between *rrnS* and the *trnM* is 436 bp.

To construct the phylogenetic tree, the complete mitogenomes from 12 geometroid species and three drepanoid species as ingroups, and two noctuoid species as outgroups were used. Bayesian inference (BI) analysis was executed using MrBayes on XSEDE (3.2.6) (https://www.phylo.org/portal2/home.action), with the Markov chain Monte Carlo analysis run for 10,000,000 generations, sampled every 1000th generation and with a burn-in of 25%. The results are shown in Figure 1. The family Uraniidae belongs to the superfamily Geometridae, which is consistent with Yang et al. (2019). Meanwhile, the closest relationship between Uraniidae and Epicopeiidae was strongly supported by Bayesian posterior probabilities values of 0.99.

**Figure 1.**
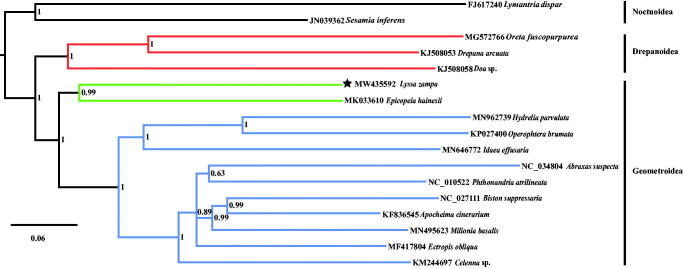
Bayesian (BI) tree of evolutionary relationships for *Lyssa zampa* based on the complete mitogenomes of 17 lepidopteran moths.

## Data Availability

The mitogenome sequence data that support the finding of this study are openly available in GenBank of NCBI at https://www.ncbi.nlm,nih.gov under the accession no. MW435592.
